# Identification of aberrantly expressed genes during aging in the mouse heart via integrated bioinformatics analysis

**DOI:** 10.1097/MD.0000000000041972

**Published:** 2025-03-28

**Authors:** Pianpian Huang, Jun Fu, Ji Hu, Yinghong Lei, Tingyu Wu, Ju Liu

**Affiliations:** a Departments of Geriatrics, Wuhan No. 1 Hospital, Wuhan, China; b Departments of Radiology, Wuhan No. 1 Hospital, Wuhan, China; c Department of Cardiology, Union Hospital, Tongji Medical College, Huazhong University of Science and Technology, Wuhan, China.

**Keywords:** bioinformatics, biomarkers, cardiovascular disease, Gene Expression Omnibus, heart aging

## Abstract

Cardiovascular disease (CVD) represents a global problem and is associated with high levels of morbidity/mortality in the elderly (>65 years old). The present study aimed to identify the key candidate genes and pathways in cardiac aging via integrated bioinformatics analysis. The GSE43556 and GSE8146 gene expression datasets were obtained from the Gene Expression Omnibus (GEO) database, and differentially expressed genes (DEGs), defined as *P* < .05 and |log fold-change (FC)| >0.5, were identified. Functional enrichment and protein-protein interaction network construction were subsequently performed. First, 142 DEGs shared between the two GEO datasets were identified. Second, biological functional enrichment analysis illustrated that these DEGs mainly participate in “inflammatory response” and “monocarboxylic acid metabolic process.” Moreover, Kyoto Encyclopedia of Genes and Genomes pathway analysis revealed that the DEGs were mainly enriched in the PI3K-Akt signaling pathway. Subsequently, the association between the expression of DEGs in the aged heart was evaluated using the Search Tool for the Retrieval of Interacting Genes database and Cytoscape software. The present study elucidated the key genes and signaling pathways associated with cardiac aging, thus improving the understanding of the molecular mechanisms underlying cardiac aging. These identified genes may be used as molecular biomarkers for the diagnosis and treatment of cardiac aging.

## 1. Introduction

Cardiovascular disease (CVD) accounts for > 30% of global deaths and is increasingly prevalent in the aging population.^[1]^ Notably, aging is considered a major independent risk factor for common heart disease, including heart failure (HF).^[2]^ During aging, the heart undergoes significant physiological and molecular changes, including cardiomyocyte hypertrophy and increased fibrosis, resulting in increased ventricular stiffness and a reduction in diastolic cardiac function, but no change in systolic function.^[3]^ Despite the growing demand for interventions, effective treatments for age-related cardiac remodeling and dysfunction have yet to be determined.^[4]^ Therefore, identifying the molecular mechanisms underlying cardiac aging is very important for diagnosis, prevention and individualized treatment.

Cardiac transcriptome analysis is a powerful approach for the analysis of gene function. Gene expression profiling techniques, alongside statistical analysis and publicly available bioinformatics tools, can be used to identify pathways, regulatory sequences and candidate genes that are implicated in human aging.^[5]^ Gene chips, as a gene detection technology, have been widely used and the corresponding data have been stored in public databases.^[6]^ The integration and reanalysis of these genomic data provides the possibility to identify biomarkers related to certain diseases. A number of studies based on a single cohort have elucidated the pathogenesis of cardiac aging based on microarray data profiles,^[7,8]^ which leads to poor reproducibility and consistency. To overcome these shortcomings, comprehensive bioinformatics methods should be combined with expression profiling technology.

In the present study, the differentially expressed genes (DEGs) between aged and young heart tissue samples were analyzed to achieve a better understanding of heart senescence. Gene Ontology (GO) and Kyoto Encyclopedia of Genes and Genomes (KEGG) enrichment analyses of the DEGs were performed, and the protein-protein interaction (PPI) network generation and module analysis of the DEGs was conducted. The aim of the present study was to identify key genes and pathways in cardiac aging using bioinformatics analysis, to subsequently explore the intrinsic mechanisms of cardiac aging, and to distinguish novel potential diagnostic and therapeutic biomarkers. The findings of the present study may provide further insight into the pathogenesis and development of cardiac aging at the molecular level.

## 2. Materials and methods

### 2.1. Datasets

The Gene Expression Omnibus (GEO; https://www.ncbi.nlm.nih.gov/geo) is a public functional genomics data repository that includes array- and sequence-based data, which is free for all users. For the present study, a data search was conducted in the GEO database. The search terms “mouse,” “aging,” “heart” and “mRNA” provided the datasets, GSE43556^[9]^ and GSE8146,^[10]^ which were found to meet the conditions. GSE43556 was based on the GPL1261 platform [Mouse430_2] Affymetrix Mouse Genome 430 2.0 Array. The GSE43556 dataset has 8 samples, including 4 aging heart samples and 4 matched young heart samples. GSE8146 was based on the GPL81 [MG_U74Av2] Affymetrix Murine Genome U74A Version 2 Array. The GSE8146 dataset consists of 20 samples, 10 of which were included in the present analysis, including 5 aged heart samples and 5 matched young heart samples.

### 2.2. Analysis of gene chip data and DEGs

Gene chip data analysis of the GEO datasets was performed in R 3.6.3 software (http://www.r-project.org). The sample size of a single data set is small and is thus prone to bias; therefore, instead of analyzing the DEGs that appear in only one dataset, a joint analysis of the two GEO datasets was performed. To make the data comparable, we first standardized the data using the surrogate variable analysis (SVA) R package for batch correction (https://www.bioconductor.org/packages/release/bioc/html/sva.html). The combined dataset was normalized performed using Limma software package (https://www.bioconductor.org/packages/release/bioc/html/limma.html). The DEGs between the aged and young hearts were detected using unpaired Student *t* test. Multiple verification was executed using the Normalize Between Arrays method. Based on factors such as the distribution of the data in our dataset, experimental design, and sample size, |logFC| >0.5 and adjusted *P* < .05 were selected as thresholds to obtain true differentially expressed genes while ensuring repeatability and robustness of the analysis. We also used FDR correction was used to ensure significance of results and reduce false positives. The ggplot2 package of the R software program was used to produce volcano plots of the DEGs. The pheatmap package of the R software program was used to produce heatmaps of the DEGs.

### 2.3. GO and KEGG pathway enrichment analyses of DEGs

The Metascape online database (https://metascape.org) is a tool for gene function classification, and includes a set of functional annotation tools for investigators to analyze the biological roles of genes, and to perform GO and KEGG pathway enrichment analyses of DEGs. A count of > 2 and *P* < .05 was considered the cutoff criteria.

### 2.4. PPI network construction and module analysis

Functional PPI analysis can be used to explain the molecular mechanism of key cell activities. The Search Tool for the Retrieval of Interacting Genes (STRING) database (https://string-db.org/) was used to obtain the PPI relationships for DEGs. Briefly, DEGs were uploaded to the STRING database and the result was visualized using Cytoscape software (http://cytoscape.org/). Furthermore, significant modules were detected through the Molecular Complex Detection (MCODE) plugin in Cytoscape (https://apps.cytoscape.org/apps/mcode) based on the constructed PPI networks. The Metascape database was adopted to analyze the GO function and KEGG pathway enrichment of the two modules with the highest scores.

### 2.5. Identification of hub genes

Hub genes are important nodes with a number of interaction partners in PPI networks, which were analyzed by Cytoscape software. In addition, the Degree, Edge Percolated Component (EPC), Eccentricity, Maximal Clique Centrality (MCC), Bottleneck and Maximum Neighborhood Component (MNC) algorithms were useful methods for selecting hub genes from PPI networks.^[11]^ The CytoHubba plug-in was installed in Cytoscape, and the Degree, EPC, Eccentricity, MCC, Bottleneck and MNC scores of all nodes in the PPI network were determined. The 20 nodes with the highest score of the 6 algorithms were selected, which were intersected. In order to increase the reliability of hub genes, overlapping genes were considered to be hub genes related to heart aging. The hub genes were further analyzed by GO and KEGG pathway enrichment analyses using the Metascape database.

## 3. Results

### 3.1. Identification of DEGs

**Figure 1.**
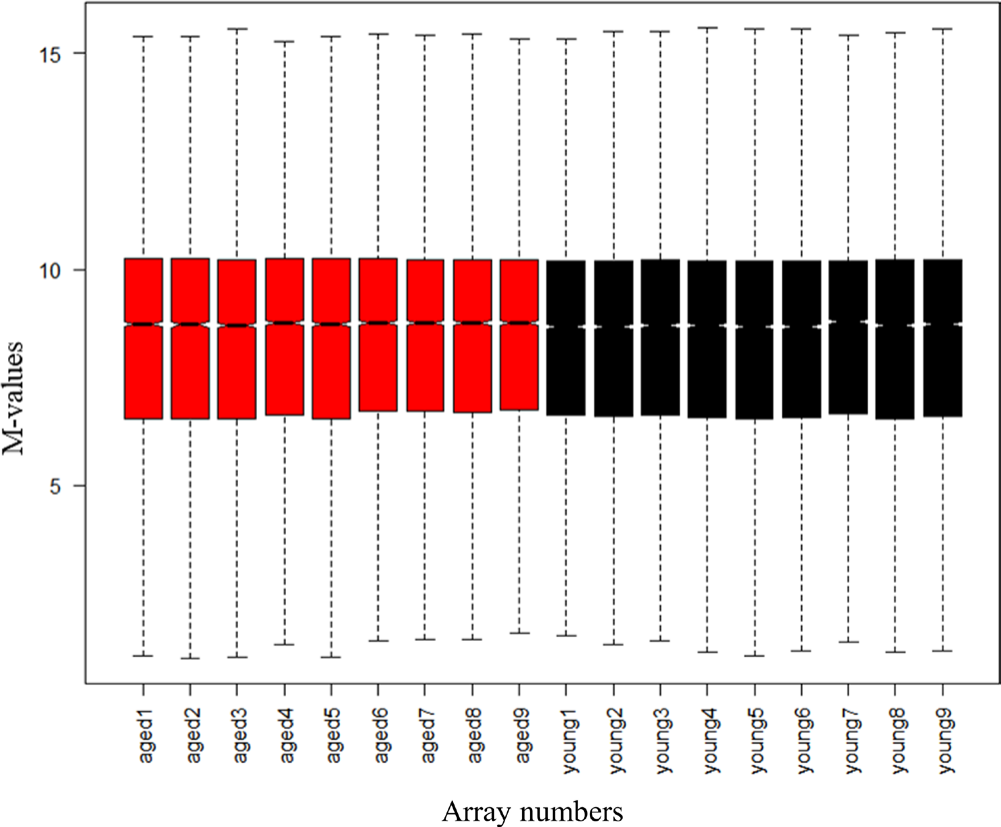
Post-batch effect correction plots. The gene expression profiles of the samples from both datasets were standardized, merged, and subjected to batch effect correction. The x-axis label represents sample symbols and the y-axis label represents the gene expression values. The horizontal lines in each column represent the mean value of gene expression.

**Figure 2.**
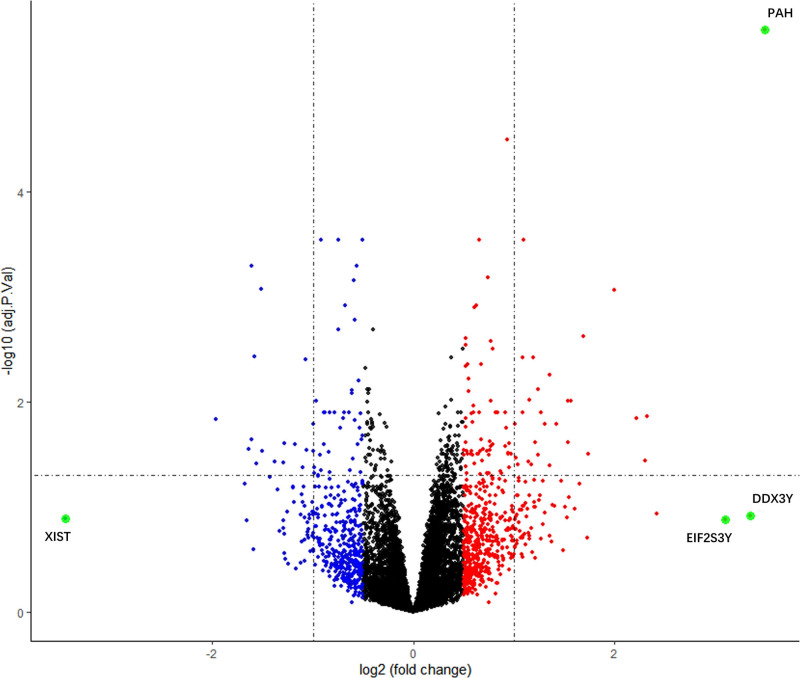
Volcano plot of the combined datasets. On the left is the volcano plot of DEGs. Red plots represent genes with |logFC|>0.5 and *P* < .05. Blue plots represent genes with |logFC| <0.5 and *P* < .05. Green plots represent genes with |logFC|>3 and *P* < .05. Black plots represent the remaining genes with no significant difference. FC = fold change.

**Figure 3.**
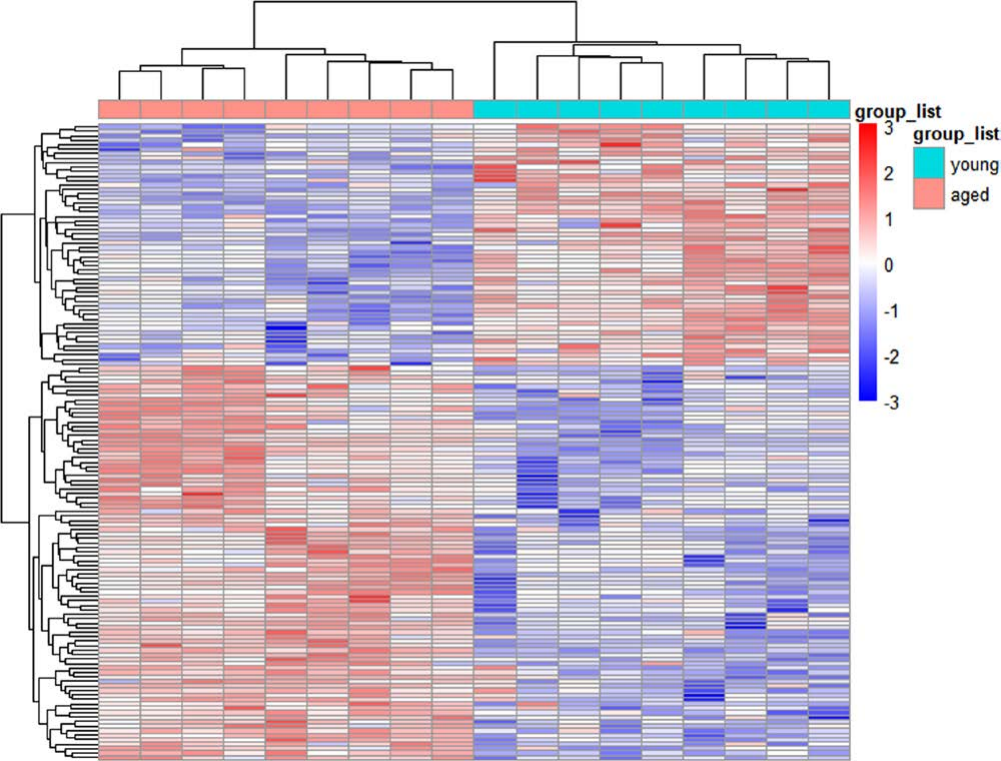
Heatmap of DEGs. Heatmap of the Upregulated and Downregulated DEGs. The heatmap showcases the expression patterns of the 88 upregulated and 54 downregulated DEGs. The color red signifies upregulation, whereas the color blue denotes downregulation. Each row represents a single gene and each column represents a sample. DEGs = differentially expressed genes.

**Figure 4.**
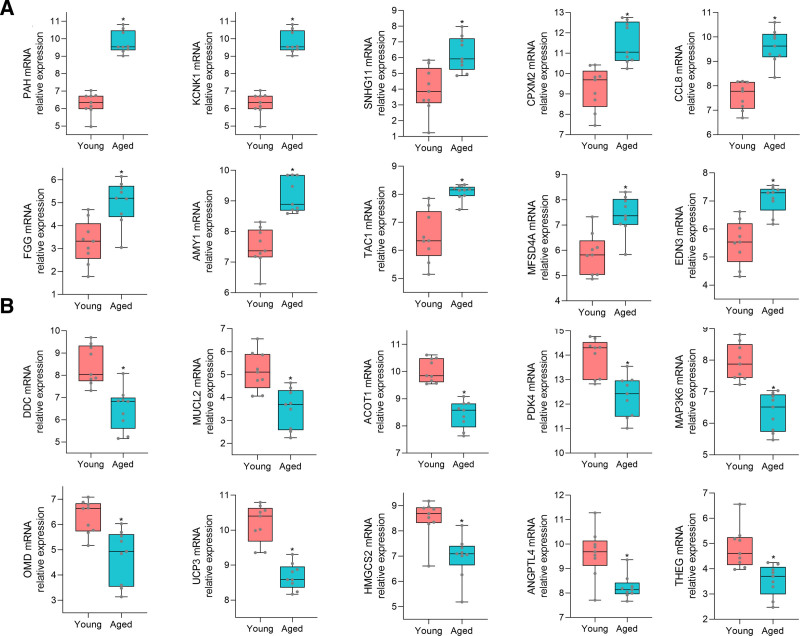
Boxplots of the top 10 upregulated genes and top 10 downregulated genes. (A) PAH, KCNK1, SNHG11, CPXM2, CCL8, FGG, AMY1, TAC1, MFSD4A, and EDN3 were significantly upregulated in aged heart tissues compared with in young heart tissues. *P < .05 vs young. (B) DDC, followed by MUCL2, ACOT1, PDK4, MAP3K6, OMD, UCP3, HMGCS2, ANGPTL4, and THEG were significantly downregulated in aged heart tissues compared with in young heart tissues. *P < .05 vs young.

Gene expression profiles of heart samples from aged mice and young control mice were obtained from the GSE43556 and GSE8146 datasets and DEGs were analyzed using R software. GSE43556 and GSE8146 were combined after batch correction. After combination, a total of 8458 genes were obtained. we conducted normalized expression analysis using limma software package, data after normalization were shown in Figure 1. Setting the cutoff criteria as *P* < .05 and |logFC|>0.5, 142 DEGs were identified, accounting for 1.68% of the total number of genes, of which 54 were down-regulated and 88 were up-regulated. A volcano plot was generated to show the DEGs between the two groups on the basis of the gene expression values (Fig. 2). The 142 DEGs were also used to generate a heatmap (Fig. 3). These genes were well clustered between aged mouse hearts and young control mouse hearts. Boxplots of the top 10 upregulated genes and top 10 downregulated genes are shown in Figure 4. The most upregulated gene was phenylalanine hydroxylase (PAH), followed by potassium channel, subfamily K, member 1 (KCNK1); small nucleolar RNA host gene 11 (SNHG11); carboxypeptidase X 2 (CPXM2); C-C motif chemokine ligand 8 (CCL8); fibrinogen γ (FGG); amylase 1 (AMY1); tachykinin 1 (TAC1); major facilitator superfamily domain containing 4A (MFSD4A); and endothelin 3 (EDN3). The most downregulated gene was dopa decarboxylase (DDC), followed by mucin-like 2 (MUCL2); acyl-CoA thioesterase 1 (ACOT1); pyruvate dehydrogenase kinase 4 (PDK4); mitogen-activated protein kinase kinase kinase 6 (MAP3K6); osteomodulin (OMD); uncoupling protein 3 (UCP3); 3-hydroxy-3-methylglutaryl-coenzyme A synthase 2 (HMGCS2); angiopoietin-like 4 (ANGPTL4); and testicular haploid expressed gene (THEG).

### 3.2. GO functional and KEGG pathway enrichment analysis of DEGs

**Figure 5.**
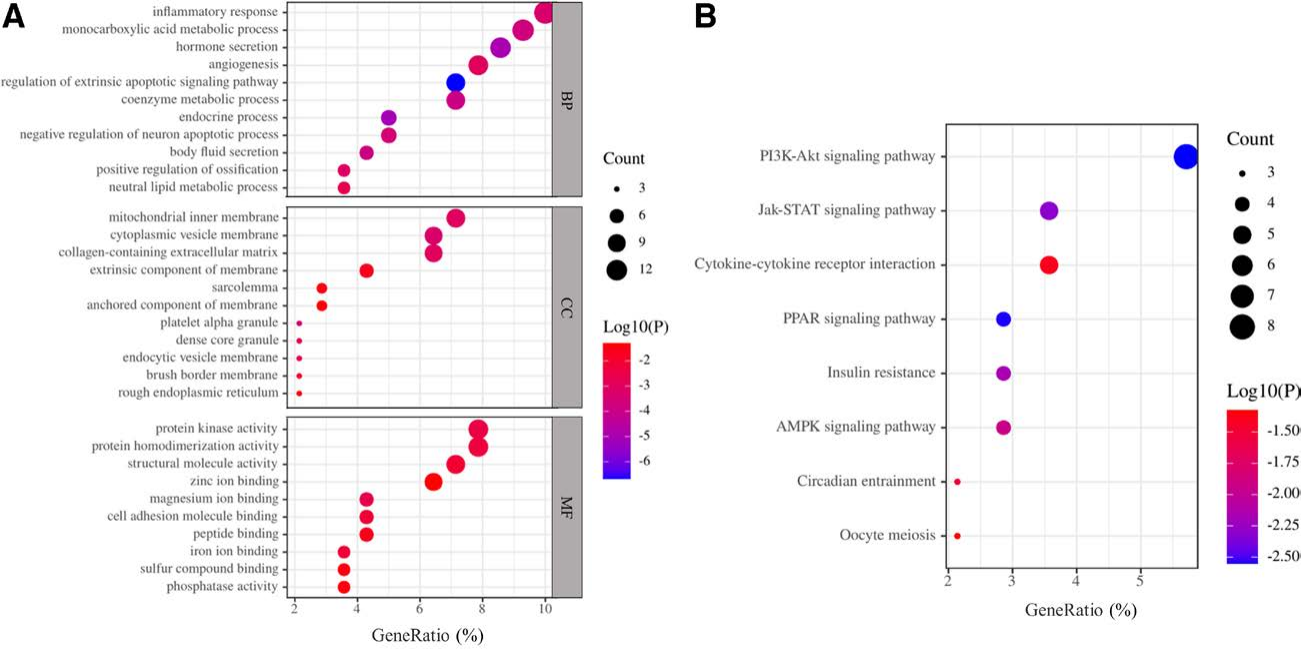
GO function and KEGG pathway enrichment analyses of DEGs. The most significantly enriched (A) GO terms and (B) KEGG pathways of DEGs. GeneRatio represents the ratio of the number of enriched DEGs and the number of annotated background genes in this pathway. The x-axis shows GeneRatio of each pathway. The y-axis refers to GO terms and KEGG pathways, respectively. BP = biological process, CC = cellular component, DEG = differentially expressed gene, GO = gene ontology, KEGG = Kyoto Encyclopedia of Genes and Genomes, MF = molecular function.

The functions of DEGs were analyzed using Metascape. The GO analysis demonstrated that, in terms of biological processes, DEGs were mainly enriched in “inflammatory response” and “monocarboxylic acid metabolic process” (Fig. 5A; Table 1). In terms of cellular components, DEGs were mainly enriched in the “mitochondrial inner membrane” and “cytoplasmic vesicle membrane” (Fig. 5A; Table 1). In terms of molecular function, DEGs were mainly enriched in “protein kinase activity” and “protein homodimerization activity” (Fig. 5A; Table 1). The KEGG pathway analysis showed that the DEGs were enriched in eight pathways; the most significant pathway was the “PI3K-Akt signaling pathway” (Fig. 5B; Table 2).

**Table 1:** GO analysis of differentially expressed genes during heart aging

Category	GO	Description	Count	%	Log10(*P*)	Log10 (*q* value)
GO biological processes	GO:0006954	inflammatory response	14	10	−3.35	−0.7
	GO:0032787	monocarboxylic acid metabolic process	13	9.29	−3.71	−0.84
	GO:0046879	hormone secretion	12	8.57	−4.96	−1.36
	GO:0001525	angiogenesis	11	7.86	−3.00	−0.48
	GO:2001236	regulation of extrinsic apoptotic signaling pathway	10	7.14	−6.67	−2.47
	GO:0006732	coenzyme metabolic process	10	7.14	−3.91	−0.92
	GO:0050886	endocrine process	7	5	−5.10	−1.38
	GO:0043524	negative regulation of neuron apoptotic process	7	5	−3.54	−0.78
	GO:0007589	body fluid secretion	6	4.29	−3.87	−0.92
	GO:0045778	positive regulation of ossification	5	3.57	−3.15	−0.57
	GO:0006638	neutral lipid metabolic process	5	3.57	−2.74	−0.32
GO cellular components	GO:0005743	mitochondrial inner membrane	10	7.14	−3.02	−0.48
	GO:0030659	cytoplasmic vesicle membrane	9	6.43	−3.29	−0.48
	GO:0062023	collagen-containing extracellular matrix	9	6.43	−2.99	−0.48
	GO:0019898	extrinsic component of membrane	6	4.29	−1.69	0
	GO:0042383	sarcolemma	4	2.86	−1.58	0
	GO:0031225	anchored component of membrane	4	2.86	−1.36	0
	GO:0031091	platelet alpha granule	3	2.14	−3.54	−0.48
	GO:0031045	dense core granule	3	2.14	−2.86	−0.42
	GO:0030666	endocytic vesicle membrane	3	2.14	−2.49	−0.15
	GO:0031526	brush border membrane	3	2.14	−1.82	0
	GO:0005791	rough endoplasmic reticulum	3	2.14	−1.57	0
GO molecular functions	GO:0004672	protein kinase activity	11	7.86	−2.47	0
	GO:0042803	protein homodimerization activity	11	7.86	−2.27	0
	GO:0005198	structural molecule activity	10	7.14	−2.04	0
	GO:0008270	zinc ion binding	9	6.43	−1.33	0
	GO:0000287	magnesium ion binding	6	4.29	−2.60	0
	GO:0050839	cell adhesion molecule binding	6	4.29	−2.14	0
	GO:0042277	peptide binding	6	4.29	−1.62	0
	GO:0005506	iron ion binding	5	3.57	−2.21	0
	GO:1901681	sulfur compound binding	5	3.57	−1.49	0
	GO:0016791	phosphatase activity	5	3.57	−1.44	0

*q* values are calculated using the Benjamini-Hochberg procedure to account for multiple testings.

GO = gene ontology.

**Table 2:** KEGG pathway analysis of differentially expressed genes during heart aging

Category	GO	Description	Count	%	Log10(*P*)	Log10（*q* values）
KEGG Pathway	mmu04151	PI3K-Akt signaling pathway	8	5.71	−2.55	−0.153109128
	mmu04630	Jak-STAT signaling pathway	5	3.57	−2.30	−0.153109128
	mmu04060	Cytokine-cytokine receptor interaction	5	3.57	−1.40	−0.089052639
	mmu03320	PPAR signaling pathway	4	2.86	−2.54	−0.079164969
	mmu04931	Insulin resistance	4	2.86	−2.16	−0.079164969
	mmu04152	AMPK signaling pathway	4	2.86	−1.94	0
	mmu04713	Circadian entrainment	3	2.14	−1.51	−0.079164969
	mmu04114	Oocyte meiosis	3	2.14	−1.33	−0.079164969

*q* values are calculated using the Benjamini-Hochberg procedure to account for multiple testings.

KEGG = Kyoto Encyclopedia of Genes and Genomes.

### 3.3. PPI analysis

**Figure 6.**
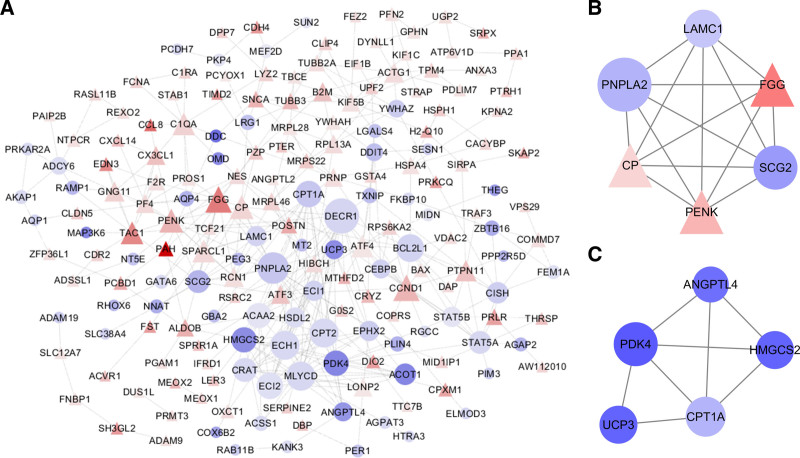
PPI network and significant modules of differentially expressed genes. (A) PPI network. Triangles indicate significantly upregulated genes; circles indicate significantly downregulated genes. Node size is positively related to combined score and node color is related to fold change. Significant modules in the PPI network with a Molecular Complex Detection score ≥ 4: (B) Module 1 (score = 6); (C) module 2 (score = 4). PPI = protein-protein interaction.

Based on information from the STRING database, a PPI network comprising 142 nodes and 128 edges was constructed using Cytoscape software (Fig. 6A). Subsequently, the networks were analyzed using the plugin MCODE with them following criteria: Node score > 4 and number of nodes > 4. Finally, two significant modules were selected (Fig. 6B and C). The genes in the two modules were enriched in the KEGG pathway “PPAR signaling pathway”; and were enriched in the GO biological processes “regulation of lipid metabolic process,” “fatty acid metabolic process,” “negative regulation of endothelial cell apoptotic process” and “response to inorganic substance” (Table 3).

**Table 3:** GO and KEGG pathway analyses of genes in the selected modules

Category	GO	Description	Count	%	Log10(*P*)	Log10（*q* values）
GO Biological Processes	GO:0019216	regulation of lipid metabolic process	4	36.36	−4.60	−1.19
	GO:2000352	negative regulation of endothelial cell apoptotic process	3	27.27	−6.27	−2.08
	GO:0006631	fatty acid metabolic process	3	27.27	−3.01	0
	GO:0010035	response to inorganic substance	3	27.27	−2.68	0
KEGG Pathway	mmu03320	PPAR signaling pathway	3	27.27	−4.94	−2.25

q values are calculated using the Benjamini-Hochberg procedure to account for multiple testings.

GO = gene ontology, KEGG = Kyoto Encyclopedia of Genes and Genomes.

### 3.4. Hub gene screening

Hub genes were detected in the network using the CytoHubba plugin, and six hub genes were identified using the six calculation methods (Degree, EPC, Bottleneck, Eccentricity, MCC and MNC). The results are shown in Table 4. The 6 most significant genes were patatin like phospholipase domain containing 2 (PNPLA2), proenkephalin (PENK), ceruloplasmin (CP), secretogranin II (SCG2), FGG and C-X3-C motif chemokine ligand 1 (CX3CL1).

**Table 4:** Hub genes analyzed by different topological algorithms in the protein-protein interaction network

Topological algorithm	Top 20 genes were ranked by score
Maximal Clique Centrality (MCC)	PNPLA2, PENK, CP, SCG2, FGG, LAMC1, PDK4, CPT1A, TAC1, ANGPTL4, GNG11, CX3CL1, BCL2L1, UCP3, HMGCS2, PTPN11, C1QA, ALDOB, B2M, PAH
Degree	PNPLA2, PENK, SCG2, CP, FGG, TAC1, PDK4, CX3CL1, BCL2L1, C1QA, CPT1A, B2M, PTPN11, CISH, LAMC1, GNG11, ATF3, UCP3, HMGCS2, ALDOB
Edge Percolated Component	PNPLA2, FGG, PENK, SCG2, CP, LAMC1, PDK4, CPT1A, TAC1, ANGPTL4, UCP3, HMGCS2, CX3CL1, GNG11, PZP, PLIN4, ALDOB, MT2, ACOT1, PAH
Maximum Neighborhood Component	PENK, CP, PNPLA2, PDK4, SCG2, FGG, TAC1, LAMC1, GNG11, BCL2L1, CPT1A, UCP3, HMGCS2, ANGPTL4, ALDOB, PAH, PTPN11, CX3CL1, CCND1, PLIN4
EcCentricity	PNPLA2, LAMC1, LRG1, G0S2, FGG, CP, SCG2, PENK, B2M, MT2, DIO2, PZP, ANGPTL4, CPT1A, PLIN4, PDK4, UCP3, ATF3, GNG11, CX3CL1
BottleNeck	SCG2, PNPLA2, PENK, ATF3, CP, CX3CL1, PTPN11, CCND1, FGG, B2M, C1QA, CISH, LRG1, PAH, NNAT, PLIN4, TAC1, MID1IP1, PRLR, THRSP
Common Genes of 6 Topological Algorithms	PNPLA2, PENK, CP, SCG2, FGG, CX3CL1

## 4. Discussion

Despite advances in current therapeutics, CVD has remained an intractable disease in recent decades. Aging is regarded as an essential disease-causing determinant of CVD.^[3]^ Therefore, identifying the etiological and molecular mechanisms underlying age-related cardiac remodeling and dysfunction is of great importance for prevention and therapy. With the rapid development of DNA microarray and high-throughput sequencing technologies, it is possible to research diseases at the gene level. DNA microarray gene expression profiling has been widely applied to explore DEGs involved in cardiac aging.^[7,8]^ The present study extracted data from the GSE43556 and GSE8146 datasets. A total of 142 DEGs were identified between aged heart tissue samples and young heart tissue samples using bioinformatics analysis. Subsequently, bioinformatics methods were utilized to explore these DEGs, including GO term enrichment, KEGG signaling pathway enrichment and PPI network construction.

The DEGs were mostly involved in inflammatory response, monocarboxylic acid metabolic process, hormone secretion and angiogenesis. This conforms to the knowledge that inflammatory response, metabolic process, hormone secretion and angiogenesis are all important mechanisms of cardiac aging.^[12–15]^ Numerous studies have demonstrated that the inflammatory response and hormone secretion serve essential roles in the prevention of heart disease.^[16,17]^ Martin et al^[18]^ reported that inhibiting the inflammatory response may reverse maladaptive remodeling in aging and HF. DEGs were also found to be involved in monocarboxylic acid metabolic process and angiogenesis. It has previously been reported that myocardial energy metabolism has an important role in maintaining the normal function of the heart, and disturbances of myocardial energy metabolism often lead to HF.^[19]^ In addition, disturbances of monocarboxylic acid, including lactate acid and pyruvate, can also lead to HF.^[20–22]^ Furthermore, aging impairs VEGF-mediated and androgen-dependent regulation of angiogenesis,^[23]^ and angiogenesis has been reported to contribute to CVD.^[24,25]^ Thus, exploring these biological functions could assist in the prediction of age-related cardiac remodeling. Subsequently, pathway analysis was performed using KEGG processes. The results indicated that all DEGs were mainly related to the PI3K-Akt signaling pathway. Numerous studies have shown that activating the PI3K-Akt signaling pathway can inhibit cardiac aging and HF,^[26–28]^ thus suggesting that the PI3K-Akt signaling pathway is an important target for the prevention and treatment of cardiac aging.

A PPI network of the DEGs with 142 nodes and 128 edges was constructed. In this network, 2 significant modules were screened depending on the degree of importance. The 2 most significant submodules of DEGs were extracted from the PPI network with MCODE scores of ≥ 4. After GO functional and KEGG pathway enrichment analyses of the DEGs in the two modules, the genes in these modules were revealed to be mainly enriched in regulation of lipid metabolic process. Metabolic alterations caused by senescence occurred at different function levels.^[29–31]^ It has been shown that in the aging heart, mitochondrial respiratory functions are affected, especially the oxidative phosphorylation enzymes and the citric acid cycle.^[29]^ A decrease in age-related fatty acid oxidation has also been observed in normal hearts.^[30]^ Furthermore, decreased lipid fluidity is associated with aging. When the lipid metabolism process is disturbed, the mitochondria in elderly hearts exhibit higher levels of lipid peroxidation and injury.^[31]^ Thus, regulating cardiac lipid metabolism may be an important approach to delay the aging of the heart. The DEGs were also enriched in the PPAR signaling pathway, which are also mainly involved in regulating glucose metabolism and lipid metabolism.^[32]^ Previous studies have revealed that PPARα delays the development of some spontaneous lesions associated with aging in the hearts of SV129 mice,^[33]^ proving the importance of this signaling pathway during cardiac aging.

The present study further analyzed the hub genes in the PPI network through six calculation methods and obtained six hub genes (PNPLA2, PENK, CP, SCG2, FGG, and CX3CL1), which may serve as potential targets for diagnosis and treatment of cardiac aging. These genes have been rarely studied in cardiac aging, and only a small number of studies have confirmed changes in the expression of CP and penk in aging tissues.^[34,35]^ PNPLA2 has been recognized as a vital triglyceride hydrolase in lipid droplets/adiposome turnover in mammalian cells.^[36]^ As previously mentioned, lipid metabolism disorders are closely related to cardiac aging, therefore PNPLA2 should be considered as an important gene associated with cardiac aging. CP is a major systemic NO oxidase activated by hypoxia, which seems to be able to exert both antioxidant and prooxidative effects.^[37]^ As we all known that NO regulates cardiac mitochondrial function in health and disease.^[38,39]^ Recent researches has reported that mitochondrial regulation of oxidative stress is a potential target for cardiac aging.^[40]^ Thus, we speculate that PENK may be involved in the process of cardiac aging by regulating mitochondrial function. SCG2 is a neuroendocrine secretory protein belonging to the chromogranin/secretogranin family.^[41]^ Cleavage of SCG2 produces the active peptide neurotropin, which induces coronary angiogenesis after myocardial infarction by enhancing VEGF signaling in endothelial cells.^[42]^ Impaired angiogenesis is an important mechanism in the remodeling of the aging heart.^[43]^ Therefore, SCG2 was predicted to be an aging-related gene. FGG has a vital role in coagulation, hemostasis and inflammation.^[44]^ There is no doubt that chronic inflammation is one of the important mechanisms of aging. Inflammatory aging plays an important role in the occurrence and development of age-related cardiovascular diseases. In addition, the link between cardiac aging and chronic inflammation has also been supported by research, which mentions that the inflammatory response caused by cardiac cell aging can trigger tissue remodeling and is a key factor in promoting the progression of aging-related heart diseases.^[45]^ From this, we speculate that FGG may be involved in cardiac aging by regulating inflammatory responses. CX3CL1 is a unique member of the CX3C chemokine subfamily. It has been reported that CX3CL1 is coupled to and acts through G protein.^[46]^ CX3CL1 is coupled to and acts through G protein,^[46]^ participating in the regulation of biological processes such as inflammation^[17]^ and angiogenesis,^[47]^ suggesting that it may be closely related to cardiac aging. These findings indicated that there is a close relationship between hub genes and cardiovascular function Thus, it was hypothesized that they may participate in aging-related heart dysfunction. Taken together, the six genes may be involved in the progression of aging-related cardiac remodeling and dysfunction.

For shortcomings, the findings of this study are primarily based on bioinformatics analysis, and the hub genes and key signaling pathways during cardiac aging have not yet been experimentally validated in aging mouse models. In future work, we will conduct experiments in aging mouse models to verify the the hub genes and key signaling pathways identified in this study during cardiac aging. More importantly, further genetic intervention research needs to be implemented to confirm their exact biological functions to determine whether they are key regulators of cardiac aging and whether they could serve as potential therapeutic targets.

## 5. Conclusion

In conclusion, through bioinformatics analysis, a series of molecular pathways related to cardiac aging were explored. In addition, six hub genes that may be related to cardiac aging, namely PNPLA2, PENK, CP, SCG2, FGG and CX3CL1, were identified from the GEO dataset. Further research is needed to elucidate the downstream mechanisms of potential targets. Understanding the mechanisms of these aging processes may result in novel breakthroughs in the prevention and treatment of cardiac aging and aging-related dysfunction.

## Acknowledgments

The authors thank the GEO databases for the availability of the data.

## Author contributions

**Conceptualization:** Pianpian Huang, Ju Liu.

**Data curation:** Pianpian Huang, Jun Fu, Ji Hu.

**Formal analysis:** Pianpian Huang, Jun Fu.

**Methodology:** Pianpian Huang, Jun Fu, Ji Hu, Yinghong Lei.

**Supervision:** Tingyu Wu, Ju Liu.

**Writing – original draft:** Pianpian Huang.

**Writing – review & editing:** Pianpian Huang.

## References

[1] Camici GG Savarese G Akhmedov A Luscher TF. Molecular mechanism of endothelial and vascular aging: implications for cardiovascular disease. Eur Heart J. 2015;36:3392–403. 26543043 10.1093/eurheartj/ehv587

[2] Virani SS Alonso A Aparicio HJ.; On behalf of the American Heart Association Council on Epidemiology and Prevention Statistics Committee and Stroke Statistics Subcommittee. Heart Disease and Stroke Statistics-2021 Update: a report from the American Heart Association. Circulation. 2021;143:e254–743. 33501848 10.1161/CIR.0000000000000950 PMC13036842

[3] Strait JB Lakatta EG. Aging-associated cardiovascular changes and their relationship to heart failure. Review. Heart Fail Clin. 2021;8:143–64. 10.1016/j.hfc.2011.08.011 PMC3223374 22108734

[4] Borlaug BA Paulus WJ. Heart failure with preserved ejection fraction: pathophysiology, diagnosis, and treatment. Eur Heart J. 2011;32:670–9. 21138935 10.1093/eurheartj/ehq426 PMC3056204

[5] Volkova M Garg R Dick S Boheler KR. Aging-associated changes in cardiac gene expression. Cardiovasc Res. 2004;66:194–204. 15820188 10.1016/j.cardiores.2004.11.016

[6] Vogelstein B Papadopoulos N Velculescu VE Zhou S Diaz LA Kinzler KW. Cancer genome landscapes. Science. 2013;339:1546–58. 23539594 10.1126/science.1235122 PMC3749880

[7] Liu C Gu X Jiang Z. Identification of novel targets for multiple myeloma through integrative approach with Monte Carlo cross-validation analysis. J Bone Oncol. 2017;8:8–12. 28856086 10.1016/j.jbo.2017.08.001 PMC5565744

[8] Ping P Guan L Ning C. WGCNA and molecular docking identify hub genes for cardiac aging. Front Cardiovasc Med. 2023;10:1146225. 37180776 10.3389/fcvm.2023.1146225 PMC10172467

[9] Boon RA Iekushi K Lechner S. MicroRNA-34a regulates cardiac ageing and function. Nature. 2013;495:107–10. 23426265 10.1038/nature11919

[10] Park SK Page GP Kim K. alpha- and gamma-Tocopherol prevent age-related transcriptional alterations in the heart and brain of mice. J Nutr. 2008;138:1010–8. 18492827 10.1093/jn/138.6.1010 PMC2768425

[11] Chin CH Chen SH Wu HH Ho C-W Ko M-T Lin C-Y. cytoHubba: identifying hub objects and sub-networks from complex interactome. BMC Syst Biol. 2014;8(Suppl 4):S11. 25521941 10.1186/1752-0509-8-S4-S11 PMC4290687

[12] Roh JD Hobson R Chaudhari V. Activin type II receptor signaling in cardiac aging and heart failure. Sci Transl Med. 2019;11:eaau8680. 30842316 10.1126/scitranslmed.aau8680 PMC7124007

[13] Xie S Xu SC Deng W Tang Q. Metabolic landscape in cardiac aging: insights into molecular biology and therapeutic implications. Signal Transduct Target Ther. 2023;8:114. 36918543 10.1038/s41392-023-01378-8 PMC10015017

[14] Mokhtari B Badalzadeh R. Mitochondria-targeted combination treatment strategy counteracts myocardial reperfusion injury of aged rats by modulating autophagy and inflammatory response. Mol Biol Rep. 2023;50:3973–83. 36829080 10.1007/s11033-023-08318-3

[15] Tang X Li PH Chen HZ. Cardiomyocyte Senescence and Cellular Communications Within Myocardial Microenvironments. Front Endocrinol (Lausanne). 2020;11:280. 32508749 10.3389/fendo.2020.00280 PMC7253644

[16] Cai S Zhao M Zhou B. Mitochondrial dysfunction in macrophages promotes inflammation and suppresses repair after myocardial infarction. J Clin Invest. 2023;133:e159498. 36480284 10.1172/JCI159498 PMC9927948

[17] Volpe M Gallo G Rubattu S. Endocrine functions of the heart: from bench to bedside. Eur Heart J. 2023;44:643–55. 36582126 10.1093/eurheartj/ehac759

[18] Martin B Gabris-Weber BA Reddy R Romero G Chattopadhyay A Salama G. Relaxin reverses inflammatory and immune signals in aged hearts. PLoS One. 2018;13:e0190935. 29346407 10.1371/journal.pone.0190935 PMC5773192

[19] Dolinsky VW Cole LK Sparagna GC Hatch GM. Cardiac mitochondrial energy metabolism in heart failure: role of cardiolipin and sirtuins. Biochim Biophys Acta. 2016;1861:1544–54. 26972373 10.1016/j.bbalip.2016.03.008

[20] Zhao G Jeoung NH Burgess SC. Overexpression of pyruvate dehydrogenase kinase 4 in heart perturbs metabolism and exacerbates calcineurin-induced cardiomyopathy. Am J Physiol Heart Circ Physiol. 2008;294:H936–43. 18083902 10.1152/ajpheart.00870.2007

[21] Evans RK Schwartz DD Gladden LB. Effect of myocardial volume overload and heart failure on lactate transport into isolated cardiac myocytes. J Appl Physiol (1985). 2003;94:1169–76. 12571142 10.1152/japplphysiol.00778.2002

[22] Johannsson E Lunde PK Heddle C. Upregulation of the cardiac monocarboxylate transporter MCT1 in a rat model of congestive heart failure. Circulation. 2001;104:729–34. 11489783 10.1161/hc3201.092286

[23] Lecce L Lam YT Lindsay LA. Aging impairs VEGF-mediated, androgen-dependent regulation of angiogenesis. Mol Endocrinol. 2014;28:1487–501. 25058601 10.1210/me.2013-1405 PMC4154238

[24] Zeng H Chen JX. Sirtuin 3, endothelial metabolic reprogramming, and heart failure with preserved ejection fraction. J Cardiovasc Pharmacol. 2019;74:315–23. 31425381 10.1097/FJC.0000000000000719 PMC6778014

[25] Sickinghe AA Korporaal SJA den Ruijter HM Kessler EL. Estrogen contributions to microvascular dysfunction evolving to heart failure with preserved ejection fraction. Front Endocrinol (Lausanne). 2019;10:442. 31333587 10.3389/fendo.2019.00442 PMC6616854

[26] Granado M Amor S Martin-Carro B. Caloric restriction attenuates aging-induced cardiac insulin resistance in male Wistar rats through activation of PI3K/Akt pathway. Nutr Metab Cardiovasc Dis. 2019;29:97–105. 30497927 10.1016/j.numecd.2018.09.005

[27] Hu WS Ting WJ Chiang WD. The heart protection effect of alcalase potato protein hydrolysate is through IGF1R-PI3K-Akt compensatory reactivation in aging rats on high fat diets. Int J Mol Sci. 2015;16:10158–72. 25950762 10.3390/ijms160510158 PMC4463638

[28] Lin CH Lin CC Ting WJ. Resveratrol enhanced FOXO3 phosphorylation via synergetic activation of SIRT1 and PI3K/Akt signaling to improve the effects of exercise in elderly rat hearts. Age (Dordr). 2014;36:9705. 25158994 10.1007/s11357-014-9705-5 PMC4453936

[29] Janero DR Hreniuk D Sharif HM. Hydroperoxide-induced oxidative stress impairs heart muscle cell carbohydrate metabolism. Am J Physiol. 1994;266(1 Pt 1):C179–88. 8304415 10.1152/ajpcell.1994.266.1.C179

[30] Kates AM Herrero P Dence C. Impact of aging on substrate metabolism by the human heart. J Am Coll Cardiol. 2003;41:293–9. 12535825 10.1016/s0735-1097(02)02714-6

[31] Cusack BJ Mushlin PS Andrejuk T Voulelis LD Olson RD. Aging alters the force-frequency relationship and toxicity of oxidative stress in rabbit heart. Life Sci. 1991;48:1769–77. 2020259 10.1016/0024-3205(91)90215-w

[32] Yamagishi S Nakamura K Matsui T. Regulation of advanced glycation end product (AGE)-receptor (RAGE) system by PPAR-gamma agonists and its implication in cardiovascular disease. Pharmacol Res. 2009;60:174–8. 19646657 10.1016/j.phrs.2009.01.006

[33] Howroyd P Swanson C Dunn C Cattley RC Corton JC. Decreased longevity and enhancement of age-dependent lesions in mice lacking the nuclear receptor peroxisome proliferator-activated receptor alpha (PPARalpha). Toxicol Pathol. 2004;32:591–9. 15603543 10.1080/01926230490515283

[34] Kim LB. Age-related changes in ceruloplasmin content in W/SSM rats. Bull Exp Biol Med. 2008;146:680–1. 19513353 10.1007/s10517-009-0365-x

[35] Caffrey JL Boluyt MO Younes A. Aging, cardiac proenkephalin mRNA and enkephalin peptides in the Fisher 344 rat. J Mol Cell Cardiol. 1994;26:701–11. 8089851 10.1006/jmcc.1994.1085

[36] Smirnova E Goldberg EB Makarova KS Lin L Brown WJ Jackson CL. ATGL has a key role in lipid droplet/adiposome degradation in mammalian cells. EMBO Rep. 2006;7:106–13. 16239926 10.1038/sj.embor.7400559 PMC1369222

[37] Fox PL Mukhopadhyay C Ehrenwald E. Structure, oxidant activity, and cardiovascular mechanisms of human ceruloplasmin. Life Sci. 1995;56:1749–58. 7739349 10.1016/0024-3205(95)00146-w

[38] Davidson SM Duchen MR. Effects of NO on mitochondrial function in cardiomyocytes: pathophysiological relevance. Cardiovasc Res. 2006;71:10–21. 16515774 10.1016/j.cardiores.2006.01.019

[39] Valdez LB Zaobornyj T Alvarez S Bustamante J Costa LE Boveris A. Heart mitochondrial nitric oxide synthase. Effects of hypoxia and aging. Mol Aspects Med. 2004;25:49–59. 15051316 10.1016/j.mam.2004.02.008

[40] Picca A Mankowski RT Burman JL. Mitochondrial quality control mechanisms as molecular targets in cardiac ageing. Nat Rev Cardiol. 2018;15:543–54. 30042431 10.1038/s41569-018-0059-z PMC6283278

[41] Fischer-Colbrie R Laslop A Kirchmair R. Secretogranin II: molecular properties, regulation of biosynthesis and processing to the neuropeptide secretoneurin. Prog Neurobiol. 1995;46:49–70. 7568909 10.1016/0301-0082(94)00060-u

[42] Albrecht-Schgoer K Schgoer W Holfeld J. The angiogenic factor secretoneurin induces coronary angiogenesis in a model of myocardial infarction by stimulation of vascular endothelial growth factor signaling in endothelial cells. Circulation. 2012;126:2491–501. 23081990 10.1161/CIRCULATIONAHA.111.076950 PMC3839617

[43] Edelberg JM Reed MJ. Aging and angiogenesis. Front Biosci. 2003;8:s1199–209. 12957863 10.2741/1166

[44] Naji DH Tan C Han F. Significant genetic association of a functional TFPI variant with circulating fibrinogen levels and coronary artery disease. Mol Genet Genomics. 2018;293:119–28. 28894953 10.1007/s00438-017-1365-6 PMC5794607

[45] Liberale L Badimon L Montecucco F. Inflammation, aging, and cardiovascular disease: JACC Review Topic of the Week. J Am Coll Cardiol. 2022;79:837–47. 35210039 10.1016/j.jacc.2021.12.017 PMC8881676

[46] Imai T Hieshima K Haskell C. Identification and molecular characterization of fractalkine receptor CX3CR1, which mediates both leukocyte migration and adhesion. Cell. 1997;91:521–30. 9390561 10.1016/s0092-8674(00)80438-9

[47] Szukiewicz D. CX3CL1 (Fractalkine)-CX3CR1 Axis in inflammation-induced angiogenesis and tumorigenesis. Int J Mol Sci. 2024;25:4679. 38731899 10.3390/ijms25094679 PMC11083509

